# Follicular thyroid carcinoma in a patient with myasthenia gravis and thymoma: a rare association

**DOI:** 10.3332/ecancer.2012.274

**Published:** 2012-10-16

**Authors:** H Ni, A Htet

**Affiliations:** 1 Department of Medicine, Melaka Manipal Medical College, Melaka 75150, Malaysia; 2 Department of Diagnostic Radiology, Naypyidaw Defence Service General Hospital, Naypyidaw, Myanmar

**Keywords:** *myasthenia gravis*, *thymoma*, *follicular carcinoma of thyroid*, *thyroid carcinoma*

## Abstract

Myasthenia gravis (MG) is an autoantibody-mediated disorder affecting the neuromuscular junction causing characteristic fatigable muscle weakness. Though it can be associated with tumours of the thymus as well as thyroid disorders, it is rare for both to coexist. The exact prevalence of thyroid carcinoma in MG with thymoma is not known but only about a dozen cases have been reported in the literature. We report a case of a 38-year-old Myanmar lady who presented with weakness and breathlessness due to MG with neck swelling. On examination, she had fatigable proximal muscle weakness and thyroid enlargement with no obvious features of hyperthyroidism. Mediastinal widening and an enlarged thyroid gland were noted on her chest X-ray and chest CT. A subtotal thyroidectomy and thymectomy were done. The histology showed follicular carcinoma of the thyroid and benign thymoma. The majority of the reported cases of thyroid carcinoma in association with MG were papillary carcinoma. Follicular carcinoma thyroid associated with MG has not yet been reported in the literature.

## List of abbreviations

MG: Myasthenia gravis
CT: Computed tomography
CXR: Chest X-ray
ECG: Electrocardiogram
ESR: Erythrocyte sedimentation rate
TSH: Thyroid stimulating hormone
T4: Thyroxine
ICU: Intensive care unit

## Introduction

Myasthenia gravis (MG) is an autoimmune disorder associated with other autoimmune conditions, most commonly thyroid disorders among which autoimmune thyroid disease is the most frequent [1]. Thymic gland abnormalities are also common in MG, with thymic hyperplasia most common, followed by thymoma [2]. There are also reported cases of thyroid carcinoma in association with MG. However, it is rare for both thymic tumours and thyroid carcinoma to coexist. In this case, we report a case of MG associated with thymoma and thyroid carcinoma.

## Case report

A 38-year-old Myanmar lady was diagnosed with MG (Osserman Grade III) clinically when she presented to a district hospital with a five-month history of drooping eyelids towards the end of the day followed by proximal muscle weakness. She had also experienced difficulty in swallowing and chewing for three months. Her condition improved with pyridostigmine bromide for only one month, after which she noticed the weakness again and presented to us with progressive breathlessness for 15 days. There were no sensory symptoms, muscle tenderness, or sphincter involvement.

She was aware of swelling in her neck for the past four years but did not experience symptoms of thyrotoxicosis. She did not seek medical consultation for her neck swelling in these years. One month ago, her thyroid function tests done at the district hospital revealed free thyroxine 108.35 nmol/l which was normal, TSH 0.001 mIU/ml. So she was given low-dose carbimazole for subclinical hyperthyroidism. She refused irradiation to the head and neck.

Physical examination showed fatigable weakness of proximal muscles but no ptosis. There was mild exophthalmos in the right eye but no ophthalmoplegia noted in both eyes. Apart from fine tremors of the hands, there were no other features of hyperthyroidism. The thyroid gland was diffusely enlarged, firm in consistency with no retrosternal extension, thrills, or bruits. Cervical lymph nodes were not enlarged, and carotid pulse was palpable bilaterally.

**Figure 1:**
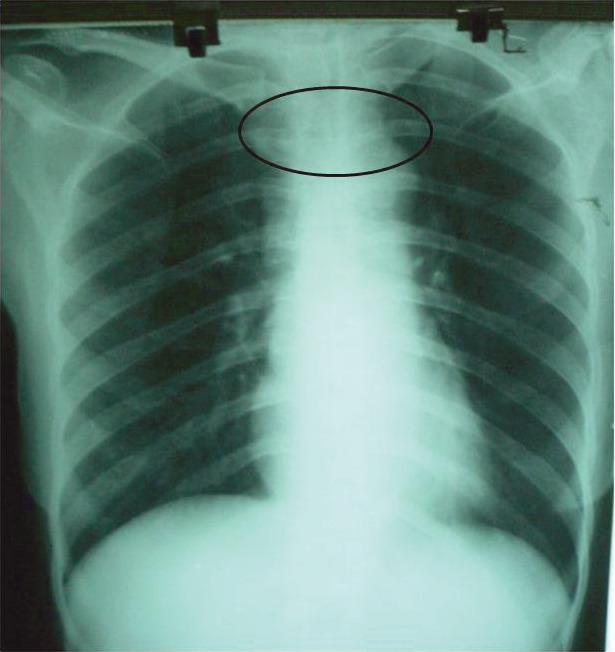
CXR. Widened mediastinum.

On admission to our unit, she was treated with pyridostigmine 30 mg four times daily and prednisolone 20 mg once daily with continuation of anti-thyroid drug. Investigations in our unit showed hypochromic microcytic anaemia (haemoglobin of 9.6%) with normal white cells and platelets. Blood urea, serum creatinine, electrolytes, cholesterol, and ESR were normal. ECG showed sinus rhythm, and mediastinal widening was noted on CXR (Figure 1). Repeat thyroid function tests in our unit revealed normal free T4 0.9 nmol/l and normal TSH 1.1 mIU/ml.

**Figure 2:**
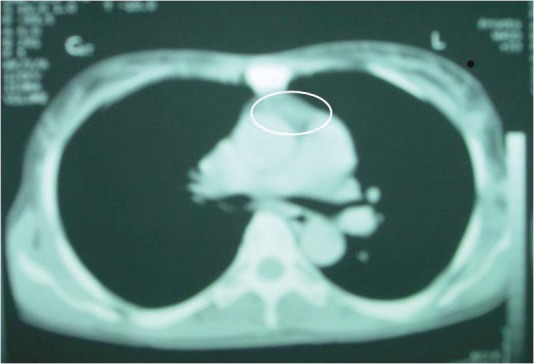
CT chest. Anterior mediastinal mass with minimal contrast enhancement.

**Figure 3:**
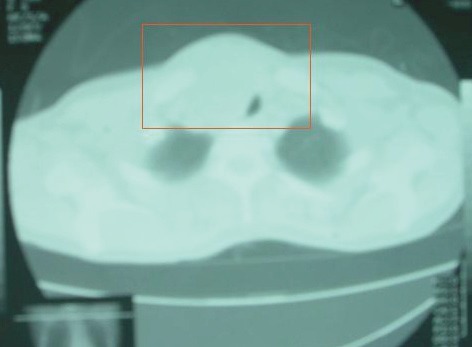
CT chest. Thyroid gland enlargement (right) with inhomogeneous contrast enhancement.

There was an anterior mediastinal mass with minimal contrast enhancement (Figure 2) and thyroid gland enlargement which was more prominent on the right side with inhomogeneous contrast enhancement on the CT scan of the chest (Figure 3).

On the fifth day of admission, she went into myasthenic crisis and was put on a ventilator in ICU for two days. After five days in ICU, her condition improved. However, she developed another crisis six days later and was put on the ventilator for a second time, and the pyridostigmine dose was doubled.

One month later, she was operated on by the thoracic surgical team after correction of anaemia. The operative findings were thymus gland enlargement with thymoma at right lower pole. The thyroid gland was also enlarged, with the right lobe measuring 5 × 5 × 4 cm, firm in consistency with increased vascularity. The cut surface appeared to be malignancy. The left lobe was normal. The regional lymph nodes were not enlarged.

Thymectomy and subtotal thyroidectomy was done. The post-operative period was uneventful with improvement of symptoms.

The gross features of her pathological report revealed nodular thyroid tissue measuring 8 × 3 × 5 cm and a satellite nodule. Serial sections showed diffusely infiltrating greyish white tumour. Another piece was a partially cystic, multinodular greyish white thymus mass measuring 6 × 3 × 2 cm.

The microscopic features of cut sections of thyroid showed circumscribed partly encapsulated nodular tumour composed of closely packed large and small colloid follicles. These follicles were lined by regular cuboidal to columnar cells with multiple layers in some areas. Nuclear pleomorphism, areas of haemorrhage, and capsular invasion were seen. Normal thyroid tissues were absent. A similar histology was noted in the satellite nodule as well. All the histological features were consistent with follicular carcinoma of thyroid.

Histology of thymus showed well-encapsulated tumour with prominent cystic degeneration. Solid areas were composed of epithelial round to oval thymocytes with central vesicular nuclei and eosinophilic cytoplasm. Nuclear pleomorphism and mitoses were absent. There was no invasion of capsule or adjacent structures. Lymphocytes were seen. The impression was thymoma with cystic degeneration, histologically benign.

A recheck of the thyroid function tests 20 days after the operation revealed TSH 22 mIU/ml, corrected calcium 8.8 mg/dl, and phosphate 2.4 mg/dl. Thus, thyroxine replacement was started at the dose of 50 μg once daily. Serum thyroglobulin levels were not elevated, and radio iodine uptake was also normal. At the next follow-up visit, there was no more muscle weakness or shortness of breath.

## Discussion

Myasthenia gravis (MG) is an autoimmune disorder characterized by abnormal fatigable weakness of muscles due to auto-antibodies directed against nicotinic acetylcholine receptors located on the post-synaptic membrane at neuromuscular junction of skeletal muscles [1].

Thymic abnormalities are common in MG and seen in approximately 75%. Thymoma occurs in nearly 15% of these, whereas thymic hyperplasia with germinal centre formation accounts for 85% [2]. The first case of thymic tumour associated with MG was reported in 1901 by Weigert. Microscopically, predominance of small round cells were noted in benign thymoma associated with MG, whereas entirely epithelial cells are present in thymomas not associated with myasthenia [3]. In the present case of thymoma in MG, there were round to oval thymocytes on the histology.

Autoimmune diseases are common in MG, seen in approximately 13% of patients. Among the associated autoimmune disorders, thyroid disease is the most frequent [4, 5]. Hyperthyroidism is the most common thyroid disorder seen in MG patients, the incidence of which is reported to be 52 per 1,000 in MG. Furthermore, increase in thymus germinal centres is seen in autoimmune hyperthyroidism. Thus, hyperthyroidism can increase thymic size and usually worsen MG [6]. The presence of acetylcholine receptor antibodies is more likely associated with thymoma, thymic hyperplasia, or thyroid disease [7]. In our patient, the initial thyroid function results showed subclinical hyperthyroidism.

Other thyroid abnormalities reported in association with MG are thyroiditis [8–11], autonomously functioning thyroid nodule [12, 13]. The first reported case of thyroid carcinoma in MG in English language was in 1983 [6], where papillary carcinoma of thyroid was detected in a 65-year-old woman with MG and thymoma. Other reports of thyroid carcinoma in MG were occult carcinoma with cervical lymph node metastases [14] and papillary carcinoma of thyroid presenting with myasthenic crisis post thyroidectomy [15]. Until now, there are a number of reported cases of thyroid carcinoma in association with MG around the world in various languages [16–20], most of which is from Japan. Histology of the reported cases of thyroid carcinoma in association with MG was papillary carcinoma [6, 15, 19, 20] with or without metastases. In the present case, the histology of the thyroid gland was consistent with follicular carcinoma of thyroid. We cannot find a case of follicular carcinoma in association with MG in the English language literature.

The exact mechanism of papillary carcinoma in MG is not known but the presence of anti-thyroid antibodies in MG, which is seen in one fourth of MG patients, might play a role [6]. However, further reports and studies are needed to establish the association between thyroid carcinoma and MG.
